# Noninvasive Mechanical Ventilation Is a Promising Way to Improve Lung Cancer Radiation Therapy

**DOI:** 10.1016/j.adro.2024.101679

**Published:** 2024-11-16

**Authors:** Johannes K. Veldman-Landegent, Zdenko van Kesteren, Mike J. Parkes, Markus F. Stevens, Joost G. van den Aardweg, Edith M.T. Dieleman, Eva Versteijne, Geertjan van Tienhoven, Arjan Bel, Irma W.E.M. van Dijk

**Affiliations:** a Department of Radiation Oncology, Amsterdam UMC location Free University, Amsterdam, The Netherlands; b Cancer Center Amsterdam, Treatment and Quality of Life, Amsterdam, The Netherlands; c Department of Anesthesiology, Amsterdam UMC location University of Amsterdam, Amsterdam, The Netherlands; d Department of Pulmonology, Amsterdam UMC location University of Amsterdam, Amsterdam, The Netherlands

## Abstract

**Purpose:**

Accurate radiation therapy (RT) for lung cancer is challenging because of the respiratory motion of the tumor and surrounding organs at risk. Recently, non-invasive mechanical ventilation (NIMV) has been investigated as a novel respiratory motion management strategy. Using NIMV, respiratory motion can be minimized, while a larger lung volume yields less overall lung dose. The purpose of this study was to determine the potential benefit of NIMV to improve lung cancer RT using magnetic resonance imaging (MRI) data of healthy volunteers.

**Methods and Materials:**

Twelve healthy volunteers practiced NIMV at 60 breaths per minute (NIMV_60_) with added positive end-expiratory pressure (PEEP) in 2 sessions and subsequently underwent NIMV_60_ in 2 MRI sessions. We acquired single-slice sagittal 2-dimensional MRI images at 2.6 Hz for 6 minutes during free breathing and NIMV_60_. We quantified the motion of all visible cross-sections of lung arteries, as a surrogate for lung tumors, in cranio-caudal and anterior-posterior directions using deformable image registration, distinguishing between 4 quadrants in the lungs (posterior-cranial, posterior-caudal, anterior-caudal, and anterior-cranial). Also, we analyzed average lung area, as a surrogate for lung volume, on the sagittal images using automatic segmentation.

**Results:**

All volunteers were successfully trained to be ventilated with NIMV_60_, and completed all sessions. The reduction of the median lung artery motion in each of the quadrants varied from 61% to 67% (from 10.7-29.9 to 3.8-11.7 mm) in cranio-caudal direction and from 51% to 68% (from 8.0-13.7 to 3.0-5.1 mm) in anterior-posterior direction using NIMV_60_. NIMV_60_ increased the sagittal lung area by 35% compared with free breathing.

**Conclusions:**

NIMV_60_ with added PEEP is a promising way to improve lung cancer RT because of reduced respiratory motion and increased lung area compared with free breathing.

## Introduction

Radiation treatment of lung cancer can result in excessive radiation dose to nearby organs at risk (OARs), eg, the heart, esophagus, and lungs, resulting in an increased risk of acute and late radiation-associated toxicity.^1^ Mean lung dose and the volume of the lungs receiving at least 20 Gy are both known risk factors of radiation-induced lung toxicity.^2^

Accurate radiation therapy (RT) for lung cancer is challenging because of the respiratory motion of the tumor and surrounding OARs. Respiratory motion management strategies that have been applied in lung cancer RT involve using an internal target volume (ITV), gating, tracking, and breath-holding.^3^ An ITV encompasses the location of the gross tumor volume (GTV) over the full respiratory cycle.^4^ Any expansion beyond the GTV results in increased radiation exposure of OARs. Strategies reducing expansions, such as gating, involve radiation delivery only during particular phases of the respiratory cycle, hence decreasing the irradiated volume. End-expiration gating reduces the target volume, but the reduced lung volume at end-expiration results in a minor decrease in mean lung dose compared with an ITV strategy while increasing the required treatment time.^5^ Tracking involves continuous recording of the tumor position and adapting the treatment beam accordingly. Accurate tracking for lung cancer RT either requires invasive placement of fiducial markers or can be done without markers with > 70% accuracy on a conventional linear accelerator^6^ or higher accuracy on a magnetic resonance imaging (MRI)-guided RT system,^7^ but tracking with or without MRI guidance is not widely available.^8^ Breath-holding requires the patient to hold their breath repeatedly for 20 to 30 seconds, during which radiation is delivered. However, breath-holding depends on patient compliance^9^ and may be inaccurate because of position variation between breath-holds^10^ and motion during breath-holds.^11^

Recently, non-invasive mechanical ventilation (NIMV) has been investigated as a novel respiratory motion management strategy^12^^,^^13^ and several types of NIMV have been investigated for lung cancer RT.^14^, ^15^, ^16^ NIMV at 2 breaths per minute (brpm), mimicking repeated breath-holds, in 6 patients with lung cancer resulted in a mean planning target volume (PTV) reduction of 47.2%. NIMV at 30 brpm in 5 patients with lung cancer resulted in decreased tumor motion in 3 patients, but increased motion in 2 patients.^15^ Continuous positive airway pressure (CPAP) in 10 patients with lung cancer increased lung volume by 32% and decreased tumor motion by 5 mm.^14^ A higher ventilation frequency of 60 brpm combined with positive end-expiratory pressure (PEEP, comparable to CPAP) is also feasible^17^ and reduces respiratory motion while also increasing lung volume, shown as an increase of the corresponding sagittal lung area on MRI.^17^ As such, ventilation at 60 brpm with PEEP would be a promising motion management strategy for radiation treatments of lung cancer. However, because NIMV at 30 brpm did not result in motion reduction in 2 of 5 patients with lung cancer,^15^ the potential benefits of this promising novel ventilation strategy must first be ascertained in a cohort of healthy volunteers.

Therefore, the purpose of this study was to determine the potential of NIMV at 60 brpm with added PEEP to improve lung cancer RT using MRI data of healthy volunteers. The primary endpoint was the quantification of residual respiratory motion of all visible cross-sections of lung arteries, as a surrogate for lung tumors, on the MR images. Secondary endpoints included the quantification of inter- and intrasession variation of lung artery motion (as measures of the intersession difference in motion and the intrasession motion consistency over time) and the quantification of sagittal lung area as a surrogate for lung volume.

## Methods and Materials

### Volunteer cohort

The medical ethics committee approved this study, and this study was registered in the International Clinical Trials Registry Platform. Between March 2022 and April 2023, 12 healthy volunteers enrolled after complying with inclusion and exclusion criteria (Table E1), contra-indications for MRI (Table E2), and after giving written informed consent. None of the volunteers had any previous experience with NIMV.

### Training, subject preparation and safety

In 2 sessions, volunteers were trained to feel safe and comfortable being ventilated (i.e., for the subject to be passive and let the ventilator take over their breathing) through a face mask covering the mouth and nose in a supine position with arms above the head. We used a Hamilton T1 mechanical ventilator (Hamilton Medical AG) at a frequency of 60 brpm (NIMV_60_) with added PEEP of 15 cmH_2_O to increase lung volume throughout ventilation. During ventilation, peripheral oxygen saturation, heart rate, and end-tidal partial pressure of CO_2_ were monitored. In 2 subsequent sessions, thoracic MR images were acquired (MR1, MR2), during which also systolic blood pressure (BP) and diastolic BP were monitored. Ventilation would have been terminated if peripheral oxygen saturation fell < 94%, heart rate < 40 or > 130/min, or systolic BP < 70 or > 180 mm Hg.

### MRI acquisition and postprocessing

We acquired thoracic MR images during free breathing (FB) and NIMV_60_ using a T1-weighted 2-dimensional (2D)-cine MRI in the sagittal plane intersecting the right lung at 2.6 Hz for 6 minutes. Additionally, we acquired the position of the right hemidiaphragm prior to each slice acquisition using a 1-dimensional (1D) navigator. We chose to image and analyze the right lung because, in the left lung image and 1D, navigator quality might suffer from noise caused by heart motion. In the respiratory signal acquired with the 1D navigator, we found the position of the diaphragm that was in the middle between the outermost end-inspiration and end-expiration positions of the diaphragm. The image corresponding to this middle point (the mid-position image) was used as a reference image for subsequent deformable image registration (DIR) using RealTITracker.^18^ DIR provides deformation vector fields, showing the displacement of each pixel in the field-of-view from the reference image to each other image in the acquisition. On the reference image, we delineated all visible cross-sections of lung arteries and selected the median value from the deformation vector fields of all pixels within each delineation as a representative displacement value. We determined these median values over time, resulting in the quantification of the motion of these lung arteries in the cranio-caudal (CC) and anterior-posterior (AP) directions during FB and NIMV_60_.

### MRI analysis

**Figure 1:**
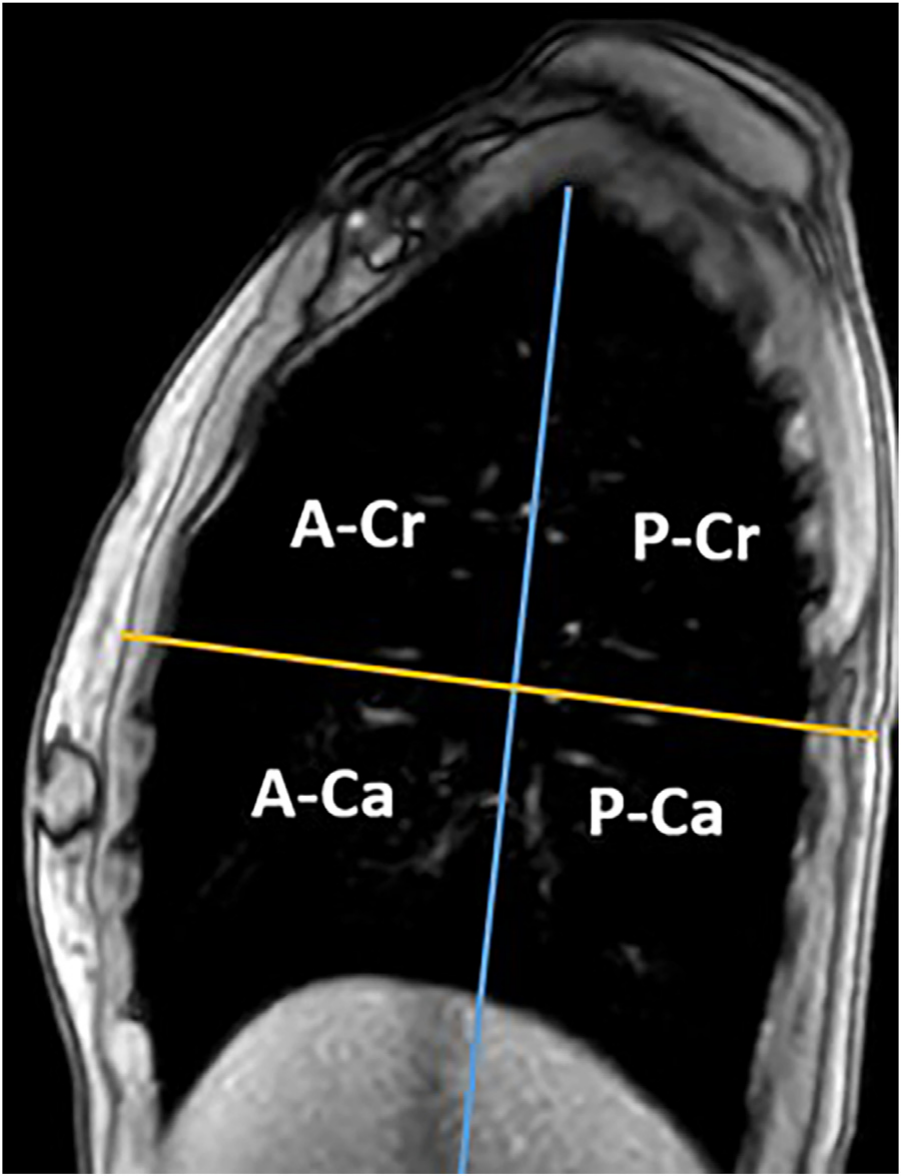
Lung quadrants are defined to sort delineated lung arteries depending on location. The vertical line runs from the lung top to halfway between the anterior and posterior ends of the diaphragm; the horizontal line runs perpendicular at half the height of the vertical line. *Abbreviations:* A-Ca = anterior-caudal; A-Cr = anterior-cranial; P-Ca = posterior-caudal; P-Cr = posterior-cranial.

For each MRI acquisition during FB and NIMV_60_, we categorized the delineated lung arteries into 4 quadrants: posterior-cranial (P-Cr), posterior-caudal (P-Ca), anterior-caudal (A-Ca), and anterior-cranial (A-Cr) (Fig. 1). This means that arteries within 1 quadrant may be positioned in different lobes. We determined the peak-to-peak motion magnitude of each lung artery in the CC- and AP-direction and grouped the results according to the 4 quadrants. Intersession motion variation was quantified by calculating the mean motion magnitude of all visible lung arteries per quadrant and volunteer in CC-direction only, and then calculating the difference between MR1 and MR2. Intrasession motion variation was calculated by dividing the 6-minute acquisition into 8 segments of 45 seconds and determining the motion magnitude of each lung artery—also in CC direction only—for each of the segments; intrasession variation was then defined as the IQR of the motion in the 8 segments. The average lung area over the 6-minute MRI was quantified based on automatic segmentations of the lung on the sagittal images using a pixel intensity filter and morphologic operations (expansions, erosions, and filling holes). All analyses were performed using in-house developed programs in MatLab (MatLab R2019b, The Math Works Inc).

### Statistical analysis

The Shapiro-Wilk test in combination with Q-Q plots showed that the data were not normally distributed. For lung artery motion and intrasession variation, we tested for statistically significant differences between MR1 and MR2; there were no significant differences between MR1 and MR2, so data from both MRI sessions were pooled. Next, we used the Mann-Whitney U test to assess differences in lung artery motion in each of the 4 quadrants during FB and NIMV_60_. For intersession and intrasession motion variation, we used the Mann-Whitney U test to determine the significance of differences in intersession and intrasession variation during FB and NIMV_60_, distinguishing between the 4 quadrants. For the lung area, we first tested for statistically significant differences between MR1 and MR2 using a Mann-Whitney U test; these differences were not significant, therefore data from both MRI sessions were pooled for FB and NIMV_60_. With the Wilcoxon sign rank test, we determined the significance of differences in lung area during FB and NIMV_60_. For all statistical tests, we used a significance level of α = 0.05. These statistical tests were performed in GraphPad Prism (GraphPad Prism 9.5.1, GraphPad Software) and SPSS (SPSS Statistics Version 28, IBM).

## Results

All volunteers (7 female and 5 male; median age 53; range, 19-65 years) were successfully trained to be ventilated with NIMV_60_ and completed all sessions. All respiratory and hemodynamic values remained within the physiological range (Figs. E1-E4), and ventilation was never terminated because none of the volunteers breached our predefined limits. Also, none of the volunteers reported any dizziness or numbness during or after NIMV_60_. For 1 volunteer, PEEP was lowered to 12 cmH_2_O for comfort. Because of failed reconstructions of the MR images, 2D cine-MRI data sets from 2 sessions were lost, resulting in 22 FB data sets and 22 NIMV_60_ data sets for analyses. In total, 255 lung arteries (P-Cr, 54; P-Ca, 100; A-Ca, 46; A-Cr, 55) were delineated in the FB images, and 223 lung arteries (P-Cr, 48; P-Ca, 92; A-Ca, 43; A-Cr, 40) in the NIMV_60_ images (Table E3).

### Lung artery motion

**Figure 2:**
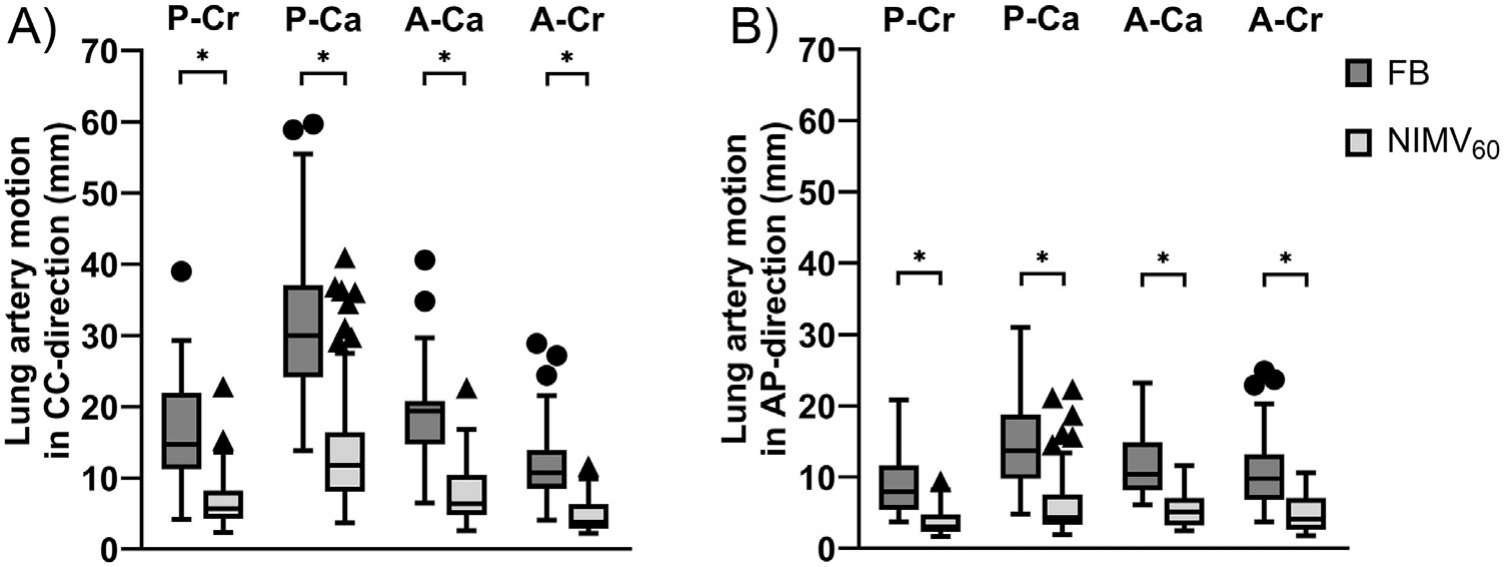
Boxplots show lung artery motion (mm) in (A) cranio-caudal direction and (B) anterior-posterior direction. NIMV_60_ with added PEEP reduces lung artery motion in both directions in all quadrants by at least 51% compared with FB. Boxes: median value and lower and higher quartiles; whiskers: lowest and highest data point within 1.5 times the IQR; circles: outliers for FB; triangles: outliers for NIMV_60_ with added PEEP. *Abbreviations:* FB = free breathing; NIMV60 = noninvasive mechanical ventilation at 60 breaths per minute; PEEP, positive end-expiratory pressure.

Figure 2 shows lung artery motion in the CC direction (Fig. 2A) and AP direction (Fig. 2B) in the 4 lung quadrants during FB and NIMV_60_. NIMV_60_ resulted in significantly (*P* < .001) smaller lung artery motion compared with FB in all quadrants. The reduction of the median lung artery motion using NIMV_60_ in each of the quadrants varied from 6.9 to 18.2 mm (61% to 67%, following [{FB-NIMV}/NIMV] × 100) in the CC direction and between 5.0 and 9.4 mm (51% and 68%) in the AP-direction.

### Intersession motion variation

**Figure 3:**
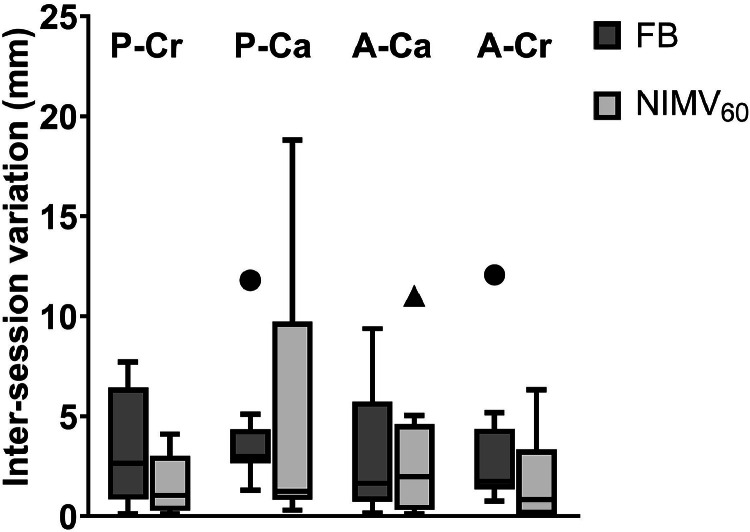
Boxplot shows the intersession variation of motion in the 4 quadrants only slightly decreases using NIMV_60_ with added PEEP compared with FB. Boxes: median value and lower and higher quartiles; whiskers: lowest and highest data point within 1.5 times the IQR; circles: outliers for FB; triangles: outliers for NIMV_60_ with added PEEP. *Abbreviations:* FB = free breathing; NIMV_60_ = noninvasive mechanical ventilation at 60 breaths per minute; PEEP = positive end-expiratory pressure.

Figure 3 shows the intersession variation of lung artery motion over 6 minutes in the CC direction between MR1 and MR2 in the 4 lung quadrants during FB and NIMV_60_. There were no significant differences between FB and NIMV_60_ for each of the quadrants. Median intersession variation increases slightly in A-Ca but reduces in all other quadrants. In P-Ca, the large value of the IQR and whisker are caused by 2 volunteers in whom the position of the delineated lung arteries within the quadrant varied considerably between MR1 and MR2. This resulted in large apparent motion differences between the MRI sessions (results without these 2 outliers are given in Fig. E5). Overall, median intersession variations in the 4 quadrants were between 1.6 and 3.0 mm for FB and between 0.8 and 2.0 mm for NIMV_60_.

### Intrasession motion variation

**Figure 4:**
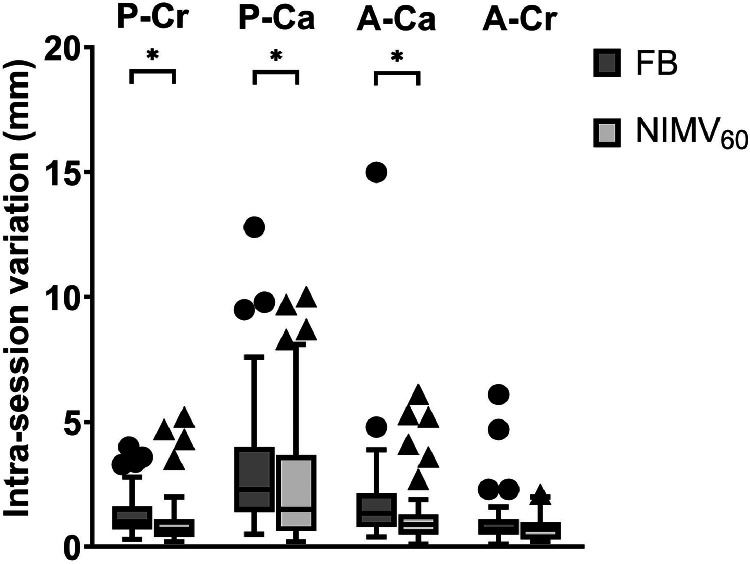
Boxplots show that the intrasession variation of lung artery motion slightly but significantly reduces in 3 of the 4 quadrants during NIMV_60_ with added PEEP compared with FB. Boxes: median value and lower and higher quartiles; whiskers: lowest and highest data point within 1.5 times the IQR; circles: outliers for FB; triangles: outliers for NIMV_60_ with added PEEP. *Abbreviations:* FB = free breathing; NIMV_60_ = noninvasive mechanical ventilation at 60 breaths per minute; PEEP = positive end-expiratory pressure.

Median intrasession variation was slightly but significantly reduced using NIMV_60_ compared with FB in 3 of the 4 quadrants (P-Cr, P-Ca, and A-Ca) and stayed equal in quadrant A-Cr (Fig. 4). Median intrasession variation was between 0.7 and 2.3 mm during FB and reduced to between 0.7 and 1.5 mm during NIMV_60_.

### Sagittal lung area

**Figure 5:**
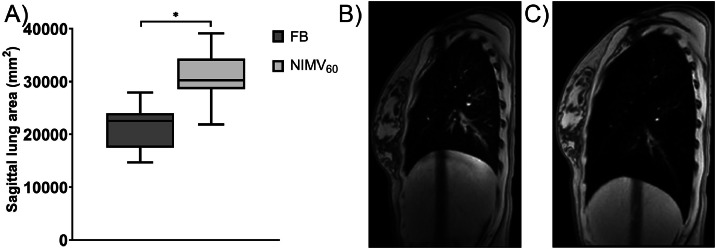
(A) Boxplots show that the sagittal lung area is significantly increased by 35% using PEEP during NIMV_60_. (B) Mid-position sagittal image during FB and (C) mid-position sagittal image during NIMV_60_ with added PEEP showing the caudal displacement of the diaphragm and increased lung area because of the applied PEEP. Boxes: median value and lower and higher quartiles; whiskers: lowest and highest data point within 1.5 times the IQR range. *Abbreviations:* FB = free breathing; NIMV_60_ = noninvasive mechanical ventilation at 60 breaths per minute; PEEP = positive end-expiratory pressure.

Figure 5 shows the mean sagittal lung area over the six6-minute MRI during FB and NIMV_60_. The mean lung area was significantly increased during NIMV_60_ through the addition of PEEP, with a median 35% larger lung area for the entire cohort and a median 41% larger (range, 18%-102%) for each individual MRI session.

## Discussion

In this study, we used MRI data from healthy volunteers and demonstrated the potential of NIMV to improve the accuracy of lung cancer RT. During NIMV_60_, median lung artery motion was significantly reduced compared with FB; intrasession variation was significantly reduced in 3 of the 4 quadrants, and intersession variation was slightly reduced. Median sagittal lung area was increased by 35% during NIMV_60_ compared with FB.

Few studies on the use of non-invasive ventilation to reduce respiratory motion have investigated the motion of lung structures or tumors. Van Ooteghem et al.^15^ found in 3 patients with lung cancer a decrease of tumor motion in the CC direction of 10%, 36%, and 48% (1.2, 2.4, and 2.8 mm, respectively) using ventilation at 30 brpm compared with FB. However, in 2 patients with lung cancer, they found an increase of motion compared with FB of 15% and 82% (0.4 and 4.9 mm, respectively).^15^ Using ventilation at 2 brpm (with 27 seconds of inflation and 5 seconds of deflation each breathing cycle), mimicking repeated deep inspiration breath-hold, Vander Veken et al.^16^ decreased tumor motion, resulting in a mean reduction of PTVs of 47.2% ± 15.3% compared with the clinical standard ITV strategy during FB. Continuous positive airway pressure (CPAP) to reduce lung tumor motion showed varying results: in 20 patients with lung cancer, mean lung tumor motion in the CC direction on the planning 4-dimensional (4D)-computed tomography (CT, 4DCT) was reduced by 5.6% (0.6 mm) using CPAP compared with FB.^19^ Contrarily, a study applying CPAP in 10 patients showed a reduction in mean lung tumor motion of 5 (SD ±8 mm), with a mean ITV reduction of 27% (95% CI, 16%-39%) compared with FB.^14^ The latter study applied higher CPAP pressures between 10 and 15 cmH_2_O, compared with 7 cmH_2_O in the first study,^19^ explaining the better results at higher CPAP pressure. In our study, we applied mechanical ventilation, in which we imposed a certain breathing pattern on the subject, whereas during CPAP, subjects still breathe spontaneously. Our NIMV_60_ showed a reduction of lung artery motion in the CC direction of 61% to 67% compared with FB, which is a more substantial reduction than found using CPAP.^14^

Lung artery motion was previously investigated in healthy volunteers, comparing non-invasive percussive ventilation at 250 to 300 brpm and ventilation-supported deep inspiration breath-hold with prolonged duration (mean 173 seconds).^20^ The authors quantified the motion pattern of lung arteries through rigid registration of MR images, incorporating interfractional and intrafractional position variations of these arteries. They created a 3-dimensional ellipsoid approximating the spatial motion distribution of these lung arteries and used the standard deviation in each direction as a representative motion magnitude parameter. Seppenwoolde et al.^21^ showed that lung tumors have heterogeneous motion patterns, and an ellipsoid shape may not be an appropriate fit to determine the motion of lung tumors or arteries. In our study, we have used DIR using RealTITracker, which can quantify heterogeneous motion patterns on 2D MRI with submillimeter accuracy.^18^

Motion quantification on MR imaging results generally in larger values compared with motion quantified on 4DCT. Mao et al.^22^ established that lung tumor motion in the CC direction on 4DCT is much smaller than motion during radiation treatment on an MR-Linac. They found that the range of lung tumor motion measured on 4DCT was only 23% of the tumor motion range on the MR-cine images during treatment. This difference in ratio between motion range on 4DCT and MRI could not be explained fully by the inherent underestimation of motion using 4DCT, which especially occurs in the presence of irregular breathing.^23^ Also, Plathow et al.^24^ reported using MRI images of patients with stage I non-small cell lung cancer or lung metastases that the lung region where a tumor is located moves significantly less than a region that does not contain a tumor. It is important to note that none of these tumors were fixed to the chest wall or pleura, which would have limited motion. We expect that lung tumor motion in patients during NIMV_60_ may be smaller than lung artery motion found in our healthy volunteers. We therefore see great potential for a prospective study to further demonstrate the feasibility and advantages of NIMV for lung cancer RT.

Because hypofractionated radiation treatments applying small margins have emerged as the clinical standard for early-stage lung cancer, it is essential that respiratory motion is reproducible. When a pretreatment 4DCT is used for treatment planning, motion during the 4DCT should be representative of motion during all radiation treatment fractions. In addition, motion magnitude should be consistent throughout treatment. Lens et al.^25^ found in 13 of 18 patients with pancreatic cancer a significant difference in tumor motion magnitude between the pretreatment 4DCT and daily cone-beam CT scans. den Boer et al.^26^ found in 10 healthy volunteers that a single estimate of diaphragm motion using 4D MRI during FB is not representative of diaphragm motion during subsequent sessions. Daily estimation of diaphragm motion improved the accuracy of the estimate, but even variation in motion magnitude during a single session occurs during FB. NIMV to regularize breathing at different frequencies can reduce intersession and intrasession variation of breathing motion.^27^, ^28^, ^29^ Our results show that intersession variation was reduced minimally, probably caused by a lack of data points in all quadrants and 2 severe outliers in the P-Ca quadrant. Eliminating these outliers resulted in a larger reduction, but still not statistically significant. Intrasession motion variation was defined as the IQR of the motion magnitudes of 8 segments of 45 seconds. This value of 45 seconds was chosen such that a cough or hiccup in one segment would still result in low intrasession variation, but coughs, hiccups, or consistent trouble with the ventilation over 2 or more segments would result in high intrasession variation. Intrasession variation was reduced minimally but significantly using NIMV_60_ compared with FB, showing that regularizing the breathing pattern with 60 brpm is achieved and motion is stable over the six-minute MRI acquisition. Applying this in an image-guided RT workflow would allow for monitoring the position of the tumor throughout treatment, allowing very tight margins.^30^

Several types of ventilation have been investigated to increase lung volume for the potential benefit of breast and lung cancer RT. Especially CPAP has been applied in patients with lung cancer to increase lung volume, aiming to decrease the dose to healthy lung tissue and the heart. Goldstein et al.^14^ achieved a 32% increase in lung volume using CPAP in 10 patients with lung cancer. This resulted in a reduction of the mean lung dose of 22% (from an average of 6.4 to 5.0 Gy) compared with FB. Also, the mean heart dose was reduced by 29% (from 6.1 to 4.2 Gy). Contrarily, Perri et al.^19^ found in patients with lung cancer a mean 8% increase in lung volume, and only a 0.1 Gy reduction in mean lung volume, unlikely to be clinically significant. This may be caused by their use of only 7 cmH_2_O CPAP, compared with 10 to 15 cmH_2_O applied by Goldstein et al.^14^. Park et al.^31^ also found an increased lung volume and a reduction in mean lung dose of 12% when using CPAP in lung cancer patients. Furthermore, increasing the CPAP pressure (from 4-7, 10, 14, and 17 cmH_2_O) resulted in a larger reduction of mean lung dose with every increase in pressure.^31^ We safely applied NIMV_60_ with PEEP at 12 to 15 cmH_2_O to increase lung volume and found a median increase in sagittal lung area as a surrogate for lung volume of 35% compared with FB. Finally, increasing lung volume using PEEP may have a beneficial effect on lung normal tissue complication probability, besides the geometric effect of reducing the mean lung dose. Namely, with increasing lung volume, the number of alveoli per irradiated volume decreases, which potentially reduces radiation-induced toxicity.

Lung artery motion in healthy volunteers may not be representative of tumor motion in patients with lung cancer. Additionally, the healthy volunteers did not suffer from respiratory comorbidities as often present in patients with lung cancer. However, our healthy volunteers were ventilated while in a supine position with arms above their heads, thus simulating a radiation treatment position. Non-invasive ventilation and CPAP have been applied in patients with lung cancer.^14^, ^15^, ^16^ Also, high-frequency ventilation at frequencies between 100 and 400 brpm has been safely and comfortably applied in healthy volunteers^32^ and a patient with lung cancer with chronic obstructive pulmonary disorder.^33^ Therefore, we expect that NIMV_60_ with added PEEP can be safely and successfully applied in most patients with lung cancer with or without respiratory comorbidities.

Reducing lung tumor motion would result in reduced ITVs, thereby decreasing the dose to the surrounding lungs, heart (in case that is close to the tumor), and other OARs, resulting in less radiation-induced toxicity. Gating allows for a reduction of PTVs, but end-expiration gating did not significantly reduce OAR dose compared with an ITV strategy while increasing treatment duration on a cone beam CT-based Linac.^5^ MRI-guided gating combined with inspiration breath-holds also allows for small PTVs (GTV + 3 mm isotropic margin) but also results in increased treatment duration, with a mean duty cycle of 69%.^34^ Goldstein et al.^14^ showed that CPAP reduced lung tumor motion by 5 mm on average, resulting in a 27% (8.4 cm^3^) decrease in ITVs and a 24% (16.7 cm^3^) decrease in PTVs. We have shown that we can reduce lung artery motion significantly and simultaneously increase lung area, and thus lung volume, using NIMV_60_ with added PEEP. Further research in patients with lung cancer is needed to thoroughly evaluate the feasibility of this technique and its potential to minimize lung tumor motion in order to reduce target volumes and subsequently, the risk of radiation-induced toxicity.

## Conclusions

In this study, we have shown in healthy volunteers that NIMV_60_ with added PEEP is a promising way to improve lung cancer RT because of reduced respiratory motion and increased lung area compared with FB. NIMV_60_ decreased lung artery motion by 61% to 67% in the CC direction and 51% to 68% in the AP direction, and increased sagittal lung area by 35%.

## Disclosures

Johannes Kornelis Veldman-Landegent reports financial support from Dutch Cancer Foundation (KWF). Michael J. Parkes reports financial support from Marie Sklodowska Cure Individual Fellowship. Arjan Bel reports research grants from General Electric, Varian Medical Systems Inc Elekta, and Karl Reiner. Zdenko van Kesteren reports a non-financial support from General Electric, Varian Medical Systems Inc, and Philips Healthcare. Irma van Dijk reports research grants from Varian Medical Systems Inc and Karl Reiner. The remaining authors do not have any relevant financial disclosures to report.

## Appendix Supplementary materials

D-24-00304 Supplementary material Image, application 1
