# Supplementary material for: Noninvasive Mechanical Ventilation Is a Promising Way to Improve Lung Cancer Radiation Therapy
Source: Adv Radiat Oncol. 2024 Nov 16;10(2):101679. doi: 10.1016/j.adro.2024.101679 (PMC11656083; doi:10.1016/j.adro.2024.101679)
Supplement: D-24-00304 Supplementary material [file mmc1.docx]

SUPPLEMENTARY MATERIAL

***Table S1: In- and exclusion criteria***

| Inclusion Criteria | | | |
| --- | --- | --- | --- |
|  | | **YES** | **NO** |
| 1. | Are you 18 years or older? | Ο | Ο |
| **Exclusion Criteria** | | | |
| Do you have or have you ever had: | | **YES** | **NO** |
| 1. | Asthma requiring medication | Ο | Ο |
| 2. | Diminished lung function | Ο | Ο |
| 3. | Heart failure | Ο | Ο |
| 4. | Epilepsy | Ο | Ο |
| 5. | High blood pressure (despite use of medication) | Ο | Ο |
| 6. | Brain abnormality, or abnormality of brain vasculature due to e.g. a stroke | Ο | Ο |
| 7. | Extreme obesity (BMI > 40) | Ο | Ο |
| 8. | Pneumothorax | Ο | Ο |
| 9. | Kidney failure | Ο | Ο |
| 10. | Claustrophobia | Ο | Ο |
| 11. | (Potentially) pregnant at this time | Ο | Ο |

***Table S2: MRI contra indications***

| **Questionnaire contra-indications for MRI** |  |  |
| --- | --- | --- |
| Have you ever had one of the following procedures where material was inserted: |  |  |
| - Pacemaker or (old) pacemaker leads? | Yes | No |
| - Implantable cardioverter defibrillator? | Yes | No |
| - Heart rhythm monitor (e.g. REVEAL)? | Yes | No |
| - Aneurysm clip in the head? | Yes | No |
| - Medicine pump (e.g. insulin)? | Yes | No |
| - Porth a cath? | Yes | No |
| - Neurostimulator? | Yes | No |
| - Artificial lens with metal clip? | Yes | No |
| - Metal ear prosthetics? | Yes | No |
| - Ear prosthetics that cannot be removed? | Yes | No |
| - Fixed metal bracket (wire behind teeth is not a problem)? | Yes | No |
| - Tissue expander? | Yes | No |
| - Overlapping stents with a total length of more than 10 cm? | Yes | No |
| - Any other implant? | Yes | No |
| - Is there a chance of other metal objects in your body (e.g. bullets, metal splinters)? | Yes | No |
| - One or more piercings? | Yes | No |
| Do you have: |  |  |
| - Trouble lying down? | Yes | No |
| - Trouble lying still? | Yes | No |
| - Any type of surgery in the last 6 weeks? | Yes | No |
| - Claustrophobia? | Yes | No |
| Are you: |  |  |
| - (former) metal worker or is there a chance of metal splinters in your eye socket? | Yes | No |
| - (potentially) pregnant? | Yes | No |

***Table S3: Number of datapoints for each motion analysis***

| **Lung artery motion and intra-session variation** | | | | |  |  |
| --- | --- | --- | --- | --- | --- | --- |
| Number of lung arteries delineated in the given quadrant | | | | | |  |
|  |  |  |  |  |  |  |
|  | Quadrant | P-Cr | P-Ca | A-Ca | A-Cr | Total |
|  | MR sessions |  |  |  |  |  |
| FB | 22 | 54 | 100 | 46 | 55 | 255 |
|  |  |  |  |  |  |  |
| NIMV₆₀ | 22 | 48 | 92 | 43 | 40 | 223 |
| Total |  | 102 | 192 | 89 | 95 |  |
|  |  |  |  |  |  |  |
| **Inter-session variation** | |  |  |  |  |  |
| Number of volunteers with at least 1 delineated artery in MR1 and MR2 in the given quadrant | | | | | | |
|  |  |  |  |  |  |  |
|  | Quadrant | P-Cr | P-Ca | A-Ca | A-Cr |  |
|  | MR sessions |  |  |  |  |  |
| FB | 22 | 10 | 10 | 8 | 10 |  |
|  |  |  |  |  |  |  |
| NIMV₆₀ | 22 | 8 | 10 | 8 | 10 |  |


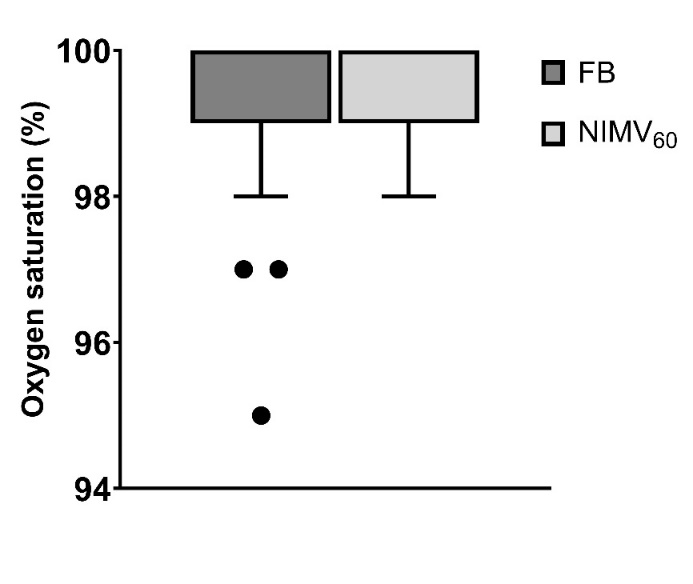


**Figure S1:** Oxygen saturation was 98% or higher in 93% of sessions for FB and always for NIMV_60_. Abbreviations: FB, free breathing; NIMV_60_, non-invasive mechanical ventilation at 60 brpm with added positive end-expiratory pressure.


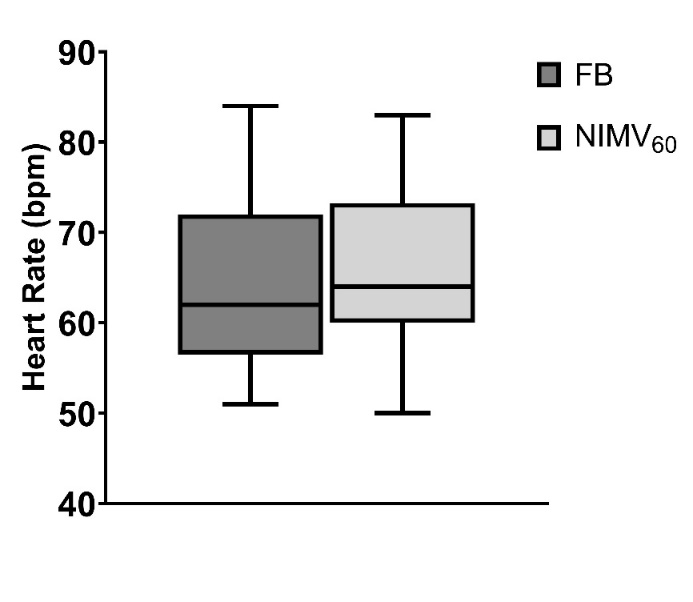


**Figure S2:** Heart rate displays only minor, clinically irrelevant changes during NIMV_60_ compared to FB. Abbreviations: FB, free breathing; NIMV_60_, non-invasive mechanical ventilation at 60 brpm with added positive end-expiratory pressure.


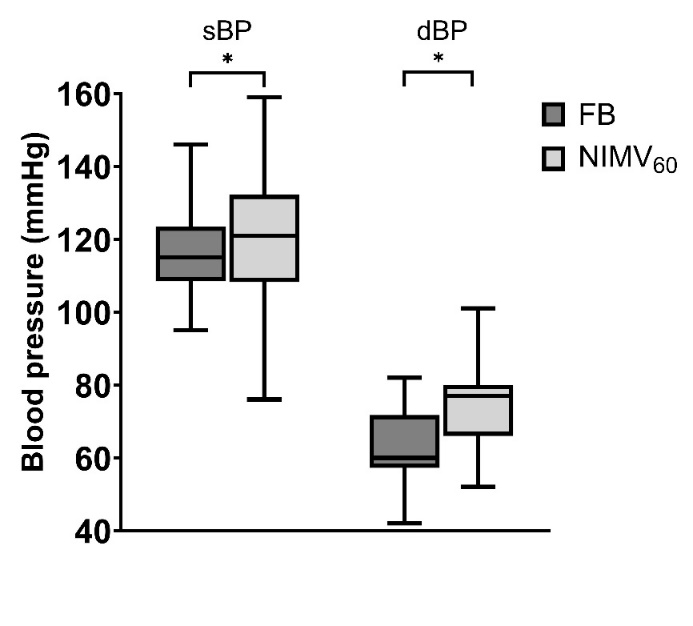


**Figure S3:** Blood pressure is significantly increased during NIMV_60_ compared to FB. However, these differences are clinically irrelevant. Abbreviations: dBP, diastolic blood pressure; FB, free breathing; NIMV_60_, non-invasive mechanical ventilation at 60 brpm with added positive end-expiratory pressure; sBP, systolic blood pressure.


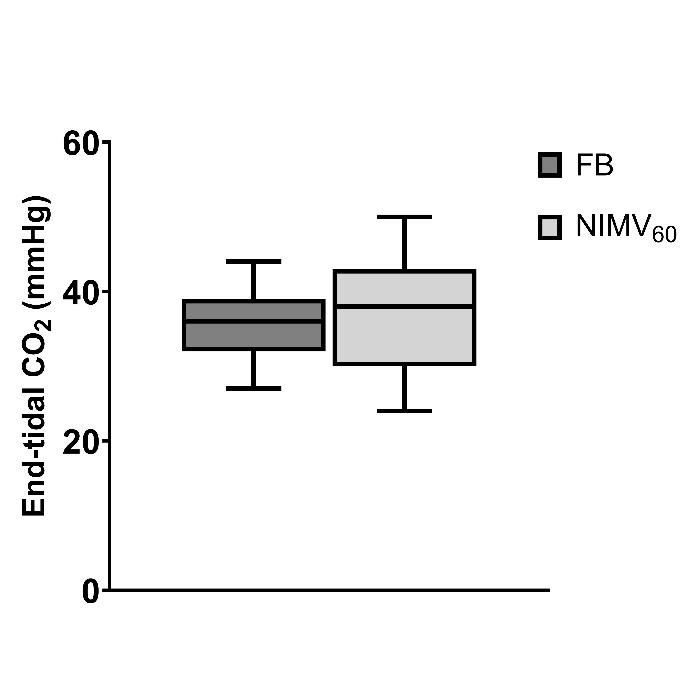


**Figure S4:** End tidal CO_2_ displays only minor, clinically irrelevant changes during NIMV_60_ compared to FB. None of the volunteers was hypocapnic during NIMV_60_. Abbreviations: FB, free breathing; NIMV_60_, non-invasive mechanical ventilation at 60 brpm with added positive end-expiratory pressure.

| 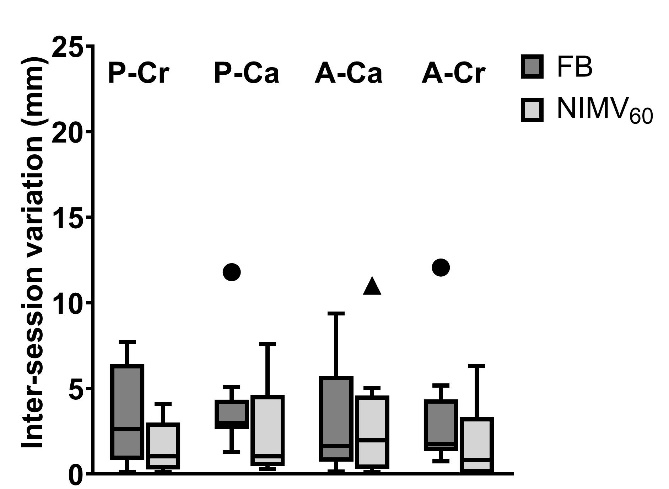 |
| --- |
| ***Figure S5:*** *Inter-session variation of motion in the four quadrants without two large outliers for the P-Ca quadrant during NIMV. Still no significant difference between FB and NIMV_60_ for this quadrant, but only just (p=0.07). Boxes: median value and lower and higher quartiles, whiskers: lowest and highest data point within 1.5 times the inter-quartile range; circles: outliers for FB; triangles: outliers for NIMV_60_. Abbreviations: FB, free breathing; NIMV_60_, non-invasive mechanical ventilation at 60 brpm with added positive end-expiratory pressure.* |
